# Exploring the Emotional Experience During Instant Messaging Among Young Adults: An Experimental Study Incorporating Physiological Correlates of Arousal

**DOI:** 10.3389/fpsyg.2022.840845

**Published:** 2022-04-04

**Authors:** Anne-Linda Camerini, Laura Marciano, Anna Maria Annoni, Alexander Ort, Serena Petrocchi

**Affiliations:** ^1^Institute of Public Health, Università della Svizzera Italiana, Lugano, Switzerland; ^2^Department of Business Economics, Health and Social Care, University of Applied Sciences and Arts of Southern Switzerland, Manno, Switzerland; ^3^Department of Health Sciences and Medicine, University of Lucerne, Lucerne, Switzerland; ^4^Faculty of Communication, Culture and Society, Università della Svizzera Italiana, Lugano, Switzerland

**Keywords:** instant messaging, emotional arousal, experiment, young adults, heart rate, electrodermal activity, physiology

## Abstract

Instant messaging (IM) is a highly diffused form of communication among younger populations, yet little is known about the emotional experience during IM. The present study aimed to investigate the emotional experience during IM by drawing on the Circumplex Model of Affect and measuring heart rate and electrodermal activity as indicators of arousal in addition to self-reported perceived emotional valence. Using an experimental design, we manipulated message latency (i.e., response after 1 min versus 7 min) and message valence (positive versus negative response). Based on data collected from 65 young adults (50% male; *M*_age_ = 23.28, *SD* = 3.75), we observed arousal as participants’ electrodermal activity levels increased from the time a fictitious peer started typing a response to the receipt of that response, especially in the delayed condition. Electrodermal activity levels also increased in both the positive and the negative message conditions. No changes were observed for heart rate. Participants’ self-report perceived emotional valence revealed that positive messages were evaluated as more pleasant and the peer as more available, while no difference in the self-report was found for message latency. These findings shed light on the emotional experience during IM by adding valuable insights on the physiological processes underlying the anticipation of social reward, but only during delayed IM exchange that can be observed in Human–Computer-Interaction.

## Introduction

The vast diffusion of digital mobile devices, especially smartphones (Silver, 2019), has enriched communication modalities, previously limited to face-to-face interactions, (electronic) mails, and phone calls, by adding the possibility to interact via Instant Messaging (IM) applications. As a form of Human–Computer-Interaction, IM took off in the mid-1990s with ICQ and AOL Instant Messenger as a web-based form of communication among the pioneers in this field. Nowadays, free-to-use, online-based IM services such as WhatsApp are leaving behind mobile short messaging services (SMS) (Preece et al., 2003). In January 2021, WhatsApp was actively used by 2 billion people worldwide (Tankovska, 2021a). In the third quarter of 2020, almost half (46%) of all WhatsApp users in the U.S. were between 15 and 35 years old (Tankovska, 2021b).

Instant messaging allows highly flexible communication between two or more communication partners, with the possibility to communicate *via* text messages, emojis, pictures, videos, memes, voice messages, and status updates. Other affordances of IM include integrated voice and video calls, which enable an easy switch between more or less media-rich communication. The affordances of IM facilitate understanding by clarifying ambiguities and promoting interaction in a timely manner (Lengel and Draft, 1984). IM communication can happen in an asynchronous (i.e., exchange of messages without an immediate response), quasi-synchronous (i.e., exchange of messages with an immediate response), or highly synchronous manner (i.e., voice or video calls), thus making IM able to integrate immediacy behaviors without interrupting ongoing activities. Synchronicity plays a crucial role in IM as it instills a sense of co-presence. When messages fly back and forth instantly, people feel like they are present together on a temporal dimension (Park and Sundar, 2015). Furthermore, IM offers a safe environment where communication partners can actively choose what to communicate and how in a more controllable way (Derks et al., 2008b). Hence, it is not surprising that major drivers of IM include the maintenance of contacts, a sense of connectedness despite physical distance, perceived social presence, perceived ease of use, enjoyment, emotional sharing, and search for social support (Wang et al., 2012; Yus, 2017).

To date, research has focused on the understanding of *what* is communicated, *how* and *when* it is communicated, and *which* are the effects of IM (e.g., Derks et al., 2008a; Al-Khawaldeh et al., 2016; Blabst and Diefenbach, 2017; Flores-Salgado and Castineira-Benitez, 2018; Matassi et al., 2019; Resende et al., 2019; Ayan, 2020; Petrocchi et al., 2020). For example, a study using Ecological Momentary Assessments to evaluate person- and situation-level factors of the responsiveness to WhatsApp messages found that individuals tend to respond more quickly in a one-to-one chat compared to group chats (Dogruel and Schnauber-Stockmann, 2021). Another exploratory survey study found that a high number of single IM chats via WhatsApp with only one communication partner was considered stressful and a waste of time (Blabst and Diefenbach, 2017). Furthermore, an experimental study on the role of synchronicity, modality, and message valence of WhatsApp communication found that positive messages, emojis, and quasi-synchronous responses within a 1-min time frame increased the perceived social presence of the communication partner and augmented the sense of social support (Petrocchi et al., 2020). To note, the cited examples relied on self-report perceptions of the IM situation and the communication partner. Self-report perceptions can be biased as participants may face difficulties when reporting on their cognitions, emotions, and behaviors (Slater, 2004; de Reuver and Bouwman, 2015). To overcome these impediments, physiological parameters such as heart rate (HR) and electrodermal activity (EDA) can serve as proxies of the underlying processes (Potter and Bolls, 2012) and, thus, provide valuable information on the emotional experience during IM. Emotional experiences are, indeed, a combination of emotional arousal, captured with, e.g., HR and EDA, and perceived valence, assessed with self-report evaluations of the communication situation. Hence, the present study wanted to fill the gap in current research on IM by incorporating HR and EDA as physiological correlates of emotional arousal in addition to self-report evaluations of IM through WhatsApp in a sample of young adults. In particular, by using an experimental design, two aspects of IM communication (i.e., synchronicity and emotional valence) were explored. The study investigated the fluctuations in emotional arousal *during* the different moments of IM communication, from the time individuals wait for a message to the actual reaction to the message. To our knowledge, this is the first study investigating the physiological correlates of IM communication in the broader context of studies on Human–Computer-Interaction.

### Psychophysiological Correlates of Mediated Communication

The study of psychophysiological correlates of emotional arousal in communication contexts has been primarily applied in research on media use and effects. Within these contexts, researchers have focused on physiological processes during TV, radio, or internet consumption (for an overview see Ravaja, 2004; Bolls et al., 2019). Less evidence is available on underlying physiological processes for mediated communication contexts, such as IM, social media use, and smartphone use. For example, Clayton et al. (2015) analyzed the effects of separating users from their smartphones. They observed increased HR and blood pressure when participants could not answer their ringing phone. Considering participants’ self-report feelings of anxiety and unpleasantness, they concluded that smartphone separation is associated with negative psychophysiological outcomes. Comparing the effects of face-to-face and online interaction, Shalom et al. (2015) found that, although HR and EDA did not significantly differ between the two forms of interaction, participants were less socially anxious, felt more in control, and perceived themselves as more successful in online interactions. In a similar vein, Iacovelli and Johnson (2012) compared the experiences of face-to-face *versus* IM communication after a stressful event. They found that the physical presence of the interlocutor in the face-to-face condition augmented the sense of perceived support and reduced HR and EDA. However, no differences between face-to-face and IM communication were found for self-report positive and negative emotions. Few studies to date explored the experiences *during* mediated communication. For example, Mauri et al. (2011) included physiological correlates to investigate the emotional experiences and absorption during Facebook use. They found that higher EDA levels correlated with lower levels of muscle tension, indicating that Facebook use can “evoke a psychophysiological state characterized by high positive valence and high arousal” (p. 723).

Researchers have developed two-dimensional models to classify emotions (see also Posner et al., 2005), including the Circumplex Model of Affect by Russell (1980). This model positions emotional reactions in a circular continuum around the dimensions of arousal (high *versus* low) and valence (pleasant *versus* unpleasant). According to the model, being upset is an unpleasant affect with moderate emotional arousal, while feeling tense is an unpleasant affect with high emotional arousal. On the contrary, feeling satisfied is a pleasant affect with moderate emotional arousal, while being excited is a pleasant affect with high emotional arousal. Arousal is captured by measuring psychophysiological changes in the body due to the activation of the Sympathetic Nervous System (SNS), which – together with the Parasympathetic Nervous System (PNS) – is part of the autonomic nervous system (Jänig, 1989). When activated, the SNS causes an acceleration of HR, i.e., increased beats per minute (BPM) or a shorter interbeat interval (IBI) (Potter and Bolls, 2012). However, HR is also determined by the PNS, where decreased HR is a sign of attention to and processing of external stimuli (Potter and Bolls, 2012). Given the dual nature of the heart, HR is thus challenging to interpret. Another psychophysiological measure frequently used to capture arousal is EDA, commonly measured in microSiemens (Critchley, 2002; Sequeira et al., 2009). EDA – also known as skin conductance or galvanic skin response – can be either tonic or phasic. It indicates the activation of the SNS only (Potter and Bolls, 2012). Phasic EDA is triggered by external stimuli that can be either pleasant (appetitive, positive) or unpleasant (aversive, negative). Whether IMs, or any stimulus causing emotional arousal, are perceived as pleasant or unpleasant can be assessed through self-report measures of the subjective experience (Dasborough et al., 2008) or through observations of behavioral reactions (Mauss and Robinson, 2009; Wolf, 2015). In the present study, we combined physiological correlates with self-report perceptions of IM communication.

### Emotional Experience of Message Latency and Valence

Considering the affordances of IM, different characteristics of the communication context should be considered when analyzing emotional experiences during IM. In particular, messages can differ with regards to their valence (e.g., positive, negative), latency (e.g., immediate, delayed), and modality [e.g., text, (m)emojis, pictures, videos]. While some evidence exists on the associations of these characteristics with social presence (Park and Sundar, 2015; Petrocchi et al., 2020), trust (Friend and Fox Hamilton, 2016; Petrocchi et al., 2020), and self-disclosure (Nguyen et al., 2011), none of the previous studies has incorporated psychophysiological correlates.

With regards to message latency, the question arises if an immediate *versus* a delayed message exchange leads to differences in emotional arousal (i.e., different changes in HR and EDA) and perceptions of the valence of the communication situation (i.e., evaluation as pleasant or unpleasant). There are two possible scenarios for arousal: People can be calm and relaxed while they wait for an incoming message. In this scenario, their HR should be unaffected and close to resting-state HR, which, in healthy adults, typically ranges from 60 to 100 BPM (Laskowski, 2020). Similarly, their EDA levels should be normal, ranging from 1 to 20 microSiemens (BIOPAC Introduction to Electrodermal Activity (EDA), 2021). The subsequent receipt of a message would then lead to an increase in HR and EDA due to emotional arousal provoked by a new stimulus. Indeed, arousal can result as a reaction to an expected answer to a previous request, especially if it is of high personal relevance. In the second scenario, people can be already aroused during waiting time because of higher stress levels accumulated as the (anticipated) response does not (yet) arrive. The situation of suspense (due to a delayed stimulus) *versus* surprise (due to an immediate stimulus) was already studied in the late 1960s in the context of film watching (e.g., Nomikos et al., 1968). Results showed that longer anticipation (i.e., suspense) produced higher stress levels by increasing EDA and HR, which reflected the activation of the SNS. The extent to which people may feel aroused during IM thus likely depends on *when* the stimulus (i.e., message) arrives and on *how much aroused* the communication partner already is. It also remains an open question whether people perceive the fact that they need to wait for a response as pleasant, e.g., as a form of excitement, or unpleasant. Qualitative research has shown that the pressure of being “always on” can evoke feelings of anguish, anxiety, and annoyance, leading some people to, for example, deactivate the Read Receipt function in WhatsApp chats (Matassi et al., 2019). The perceived pressure to respond immediately arises from the perception that the IM communication partner feels tense and angry because he/she does not receive a response in a timely manner. Since emotional experience during IM are a function of both arousal (e.g., calm vs. aroused) and the subjective perception of the communication situation (e.g., pleasant vs. unpleasant) we wanted to explore *how is the emotional experience, measured with HR and EDA levels for arousal and self-report perceptions for perceived valence, differently triggered in situations with longer waiting time for incoming messages compared to shorter waiting time (RQ1).*

When it comes to message valence, our previous experimental study revealed that positive messages increased the perception of social presence, which was associated with a more favorable evaluation of the IM situation and the communication partner. On the contrary, in the condition where participants received negative messages, the IM situation and communication partner were perceived as unpleasant (Petrocchi et al., 2020). The question remains how message valence may affect emotional arousal measured by HR and EDA. It is likely that HR and EDA levels increase in the context of negative messages due to feelings of anger and distress. However, we should not forget that the heart is dually innervated, i.e., “connected to both the PNS and the SNS which are constantly sending signals to some degree as neither system can ever be thought of as being ‘off’ *per se*” (Potter and Bolls, 2012, p. 28). Researchers suggested that humans are hardwired to allocate more attention to negative (or aversive) stimuli than to positive (or appetitive) stimuli to promote survival, resulting in a stronger decrease in HR (see Bolls et al., 2001). Thus, considering a situation where individuals send a request *via* IM and receive a negative answer, it is also likely that they experience slower HR while paying attention to the negative information. On the other hand, positive message valence is expected to produce higher HR and EDA levels, indicating an activation of the PNS and, thus, the experience of enjoyment as previously studied in the context of Facebook use (Mauri et al., 2011). Given the different possible scenarios of emotional arousal to incoming IMs characterized by positive vs. negative message valence, we were furthermore interested in *how is the emotional experience, measured with HR and EDA levels for arousal and self-report perceptions for perceived valence, differently triggered in situations where people receive negative messages compared to positive messages (RQ2).*

## Materials and Methods

### Study Design

We conducted a laboratory experiment where we manipulated message latency and valence in a one-to-one communication through the WhatsApp messenger service installed on a smartphone. The study built on a previous experiment manipulating message valence, latency, and modality, where we measured self-report perceptions of social presence and social support (Petrocchi et al., 2020). For the present experiment, we integrated emojis as an IM affordance related to modality in all the manipulated messages and focused on the differential effects of latency and valence. Participants were given a hypothetical scenario where they were asked to imagine that they were new in town and did not yet know anybody except for a colleague who gave them his/her mobile number. They were instructed to contact this peer through WhatsApp and to ask him/her for three favors: (1) give lessons in his mother tongue before an interview, (2) help with a work/study project, and (3) help with moving a sofa. We decided to repeat the experimental task for these three favors to increase ecological validity. The three favors were identified in a pilot study and used in our previous experiment. The order of the favors was kept constant. For reasons of ecological validity, we also decided to introduce the scenario of a colleague and not a good friend or family with whom participants likely have unique forms of IM exchange. To avoid gender effects (e.g., different communication aims and styles, romantic connotations), we matched the gender of the peer to the participants’ gender (Savicki and Kelley, 2000; Park and Lee, 2014). Participants did not know whether the communication partner was a real person or a computer. Participants were randomly assigned to four different experimental conditions. To manipulate message valence, participants were either in a condition where the peer replied with an empathic accurate message (i.e., positive valence), demonstrating understanding and offering help, or with an empathic inaccurate message (i.e., negative valence), demonstrating no understanding and neglecting help. Both message types included emojis emphasizing the respective valence. Emojis were selected from the emojis tracker for Twitter^1^ (Emojitracker, 2021): For positive messages, we included the “Smiling face with smiling eyes,” the “Smiling face with sunglasses,” and the “Smiling face with open mouth and smiling eyes,” and for negative messages, we included the “Angry faces” (both yellow and red) and the “Unamused face.” To manipulate message latency, the peer replied either after 1 min (i.e., synchronous condition) or after 7 min (i.e., asynchronous condition). We chose a 7-min delay to create a sufficient time lag compared to the synchronous condition while keeping the entire experimental session for the delayed condition below 60 min. The study received approval from the Institutional Review Board of the host university.

### Procedure

Participants were recruited through Facebook sponsoring, leaflets, and snowball sampling, targeting young adults between 18 and 35 years of age and fluent in Italian. People with a known medical condition and pregnant women were not eligible. When making their appointment, we asked participants to refrain from intense physical activity, alcohol, nicotine, and caffeine or at least 2 h before their appointment to limit the effects of these confounders during the psychophysiological acquisition. Prior to the start of the experimental session, participants received a brief explanation of the nature of physiological data collection and the study procedure in general. The experimenter answered any questions and concerns voiced by the participants and collected their informed consent.

**FIGURE 1:**
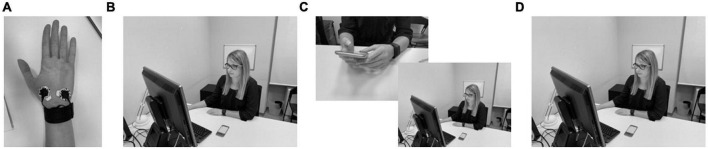
Experimental study procedure. **(A)** Preparation, **(B)** pre-questionnaire, **(C)** instant messaging task with short follow-up assessment after each task, **(D)** post-questionnnaire.

The experiment was conducted with the help of two researchers. The experimental sessions took place in a separate room within the lab of the research team, where any visual stimuli were eliminated, and the light and temperature were kept constant. To enhance ecological validity and reduce the noise in the data produced by physiological data recording devices, we used the Empatica E4 wristband (2021)^2^, which has the form of a smartwatch with embedded sensors to collect HR and EDA. Smartwatches, and digital wearables in general, are becoming an increasingly integral part of peoples’ lives (Tankovska, 2020) and are, thus, expected to produce minimal (inevitable) noise when used in experimental settings. Researcher 1 prepared participants by putting the Empatica E4 wristband on the wrist of their non-dominant hand. Validation studies showed that the E4 wristband produced reliable HR data (Schuurmans et al., 2020) but less reliable EDA data (Milstein and Gordon, 2020) compared to other physiological yet less userfriendly measurement tools. To improve measurement precision, we measured EDA by using the E4’s feature to enhance the integrated electrodes in the E4 wristband with cable extensions that can be used with Ag/AgCl electrodes (24 mm diameter), applied on the palm of the non-dominant hand (see Figure 1). The E4 also includes a Photoplethysmography (PPG) sensor to measure HR.

**FIGURE 2:**
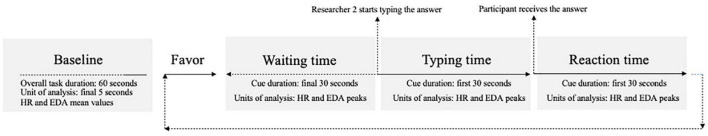
Timeline of experimental task with measurement occasions for Baseline, Waiting time, Typing time, and Reaction time.

Researcher 1 remained in the room during the entire session to supervise the data acquisition phase and protocol anomalies throughout the session, i.e., technical problems or interruptions. The experimental session began with participants answering an online pre-questionnaire accessible on a desktop screen and implemented in Qualtrics. After questionnaire completion, they were redirected to a landscape picture for 60 s. HR and EDA data collected during the final 5 s of this timeframe were later used as a baseline measure of physiological parameters. Next, participants were instructed to use an Android smartphone provided by Researcher 1 with WhatsApp installed on the home screen of the device. More specifically, they were requested to engage in a chat with a same-gender peer and ask him/her for three favors. The necessary contact details were already saved on the smartphone. Dependent on the randomly assigned experimental condition, for each of the three favors, participants received either a positive or a negative response, either after 1 min (synchronous) or 7 min (delayed). The responses were sent by Researcher 2, who was in another room and of whom the participant had no knowledge. Researcher 2 also took note of all timestamps to allow the identification of participants’ psychophysiological reactions during the three moments of interest: Waiting, Typing, and Reaction phase. In particular, the Waiting phase lasted from when Researcher 2 received the request from the participant to the time when she started typing the answer. This timeframe was either 1 or 7 min dependent on the experimental condition. We considered the final 30 s when extracting the physiological correlates. The Typing phase lasted from the time Researcher 2 started typing the answer to the time she sent the response. Again, we considered the final 30 s when extracting the physiological correlates. The Reaction phase included the time from when the participant received the answer to when he continued with the next task. We considered the first 30 s when extracting the physiological correlates. We considered a 30-s timeframe as individuals vary in the time they need to respond to external stimuli (Potter and Bolls, 2012). After each message exchange following a specific favor, participants filled out a short Qualtrics survey evaluating the experienced valence of that specific favor (task). After each task, participants saw a 1-min video with images of peaceful landscapes to come back to their baseline levels of arousal. The timeline of the experiment and all considered time points are shown in Figure 2. At the end of the experiment, participants completed a post-questionnaire assessing self-report perceptions of the entire WhatsApp communication. They received a short debriefing and a compensation equivalent to 35 US$.

### Measures

Self-report measures used in the present study stem from a pre-questionnaire to collect socio-demographic characteristics at the beginning of the experimental session and a brief evaluation of the WhatsApp communication, asking participants to rate the message exchange on a semantic differential from 1 = “unpleasant” to 7 = “pleasant,” and the peer from 1 = “unavailable” to 7 = “available.” The brief evaluation was repeated for each favor and then averaged to obtain a compound score of the perceived valence of the IM communication (*r* = 0.96).

Psychophysiological measures included BPM for HR and microSiemens for EDA. Both were automatically recorded by sensors embedded in the E4 wristband and for the timeframes described previously. For each measurement point, we calculated the deviation from baseline (DFB) to account for different resting-state HR and EDA across participants. Therefore, we subtracted the raw HR and EDA score for each recorded second from the baseline score. Next, we picked the maximum value (peak) within each timeframe of interest, i.e., waiting time, typing time, and reaction time. We did that for all three favors and averaged the DFB scores to increase ecological validity.

### Data Analysis

Because a forced-answer format was used in the questionnaires, self-report data did not include any missing values. However, missing values could occur in physiological data due to lost signals or technical interruptions during the real-time assessment of HR and EDA. Missingness was resolved in the case where the tracing could be restarted during the experimental session and later matched to real-time data collected before the interruption. We applied a listwise deletion of participants when we lost electrodes throughout the experiment, when the signal was too weak, and when the signal did not cover the necessary timeframes to extract maximum values. Also, participants with unrealistically high values attributable to electrode placement issues were removed to exclude artifacts. We performed descriptive statistics to evaluate the normal distribution of the data and to detect any outliers. We examined *z*-scores for all outcome measures and defined those above an absolute value of 3 as outliers. We further conducted χ^2^-difference tests to evaluate gender and independent samples *t*-tests to evaluate age distribution across our experimental conditions, i.e., message latency and message valence.

For our main analyses, we ran two one-way within-subjects repeated measures ANOVAs to assess participants’ emotional arousal during the different moments of the IM communication: first, by considering message latency and, second, message valence. To be able to assess the emotional experience, which – according to the Circumplex Model of Affect (Russell, 1980) – are a function of arousal and perceived valence of that arousal (i.e., positive/pleasant or negative/unpleasant), we further conducted independent samples *t*-tests to examine differences in the self-report evaluation of the communication (partner) across the experimental conditions.

### Participants

Fifteen out of 80 participants (19%) had no valid HR and EDA data for our main analysis. Thus, the analytical sample included a total of 65 young adults (*M*_age_ = 23.28, *SD* = 3.75, range 18–34 years old). Participants were balanced in gender (50% males, *n* = 32) primarily residing in Canton Ticino, Switzerland (82%, *n* = 53), high school (59%, *n* = 38) or college graduates (28%, *n* = 17). Participants reported to use their smartphone, on average, 3.5 h per day (*SD* = 1.4). The majority (80%, *n* = 52) indicated that WhatsApp, and messenger services in general, were their preferred mode of mediated communication. There were no significant differences in gender across the experimental conditions for message latency [$\chi_{\text{latency}}^{2}$ (1, *N* = 65) = 0.393, *p* = 0.531] and message valence [$\chi_{\text{valence}}^{2}$ (1, *N* = 65) = 0.393, *p* = 0.531]. Furthermore, there were no significant differences in age across the two experimental conditions for message valence [*t*_valence_ (63) = 1.719, *p* = 0.091]. However, we found a significant difference in age distribution across the two experimental conditions for message latency [*t*_latency_ (63) = −2.198, *p* = 0.032]. More precisely, participants in the synchronous condition were significantly older (*M* = 24.21, *SD* = 4.39) than participants in the delayed condition (*M* = 22.26, *SD* = 2.59). We therefore included age as a covariate in the main analyses for message latency.

## Results

The evaluation of descriptive statistics revealed a normal distribution of DFB HR, DFB EDA, and self-report assessment of the communication (partner) across the experimental conditions and the three measurement points (range of skewness –0.452 to 0.321, range of kurtosis –1.506 to 2.001). Yet, *z*-scores for DFB HR and DFB EDA above the absolute value of three indicated three outliers. The main analyses were thus run with and without these outliers. The outliers were kept when reporting our findings when the results did not change.

**FIGURE 3:**
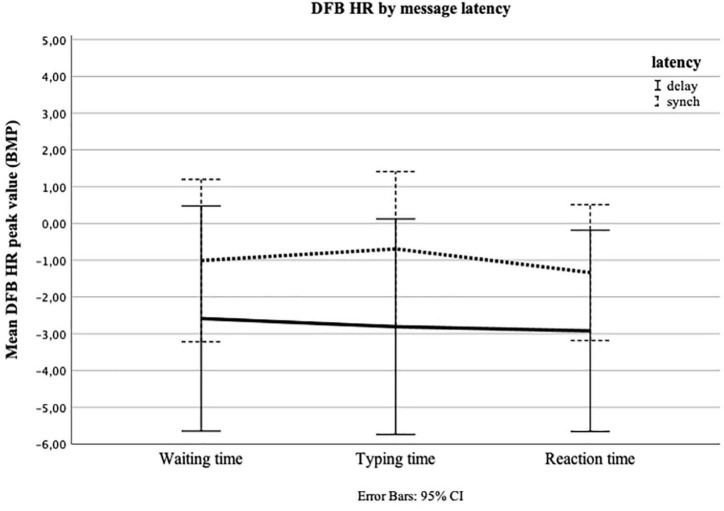
Repeated measures of heart rate (HR) in beats per minute (BPM) for Waiting time, Typing time, and Reaction time by message latency (delay = 7 min Waiting time; synch = 1 min Waiting time). The *y*-axis shows the mean peak value calculated as a deviation from the baseline measure (DFB) for the final 30 s of Waiting time, the first 30 s of Typing time, and the first 30 s of Reaction time.

**FIGURE 4:**
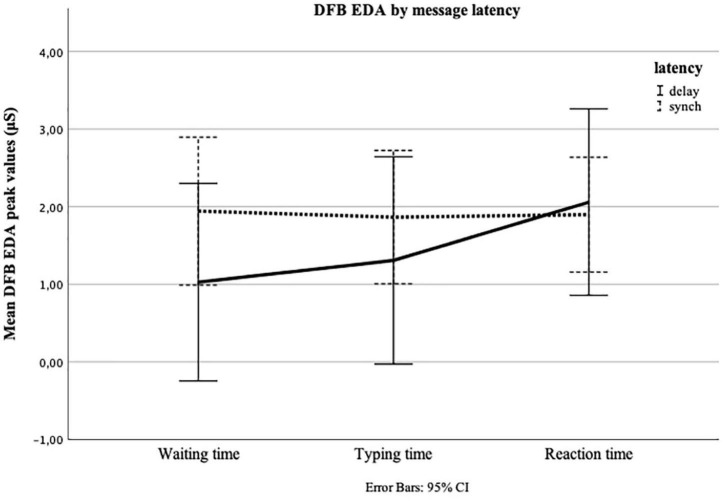
Repeated measures of electrodermal activity (EDA) in microSiemens (μS) for Waiting time, Typing time, and Reaction time by message latency (delay = 7 min Waiting time; synch = 1 min Waiting time). The *y*-axis shows the mean peak value calculated as a deviation from the baseline measure (DFB) for the final 30 s of Waiting time, the first 30 s of Typing time, and the first 30 s of Reaction time.

The main analyses included four one-way repeated-measures ANOVAs with one between-factor (message latency or valence). In the first ANOVA, we looked at the development of DFB HR, taking into consideration message latency. Since the test of sphericity revealed a violation (Grenhouse–Geisser epsilon = 0.75), we used the Huynh-Feldt correction (Haverkamp and Beauducel, 2017). The test for within-subjects effects showed that, across both conditions, i.e., synchronous and delayed, HR did not change significantly between Waiting, Typing, and Reaction time; [*F*(2,62) = 1.073, *p* = 0.333, $\eta_{\text{p}}^{2}$ = 0.017]. Neither was there a significant time × experimental group interaction [*F*(2,62) = 0.348, *p* = 0.657, $\eta_{\text{p}}^{2}$ = 0.006] (Figure 3). We repeated the ANOVA for DFB EDA. The test of within-subjects effects showed that, across both conditions, i.e., synchronous and delayed, EDA did not change significantly over time [*F*(2,62) = 0.261, *p* = 0.694, $\eta_{\text{p}}^{2}$ = 0.004]. However, we found a significant time × experimental group interaction [*F*(2,62) = 6.294, *p* = 0.007, $\eta_{\text{p}}^{2}$ = 0.092]. The test of within-subjects contrasts revealed a significant linear trend in time, dependent on message latency [*F*(1,63) = 7.025, *p* = 0.01, $\eta_{\text{p}}^{2}$ = 0.102]. The examination of the estimated marginal means (Figure 4) indicated an increase in EDA, especially between Typing time and Reaction time, in the delayed condition.

**FIGURE 5:**
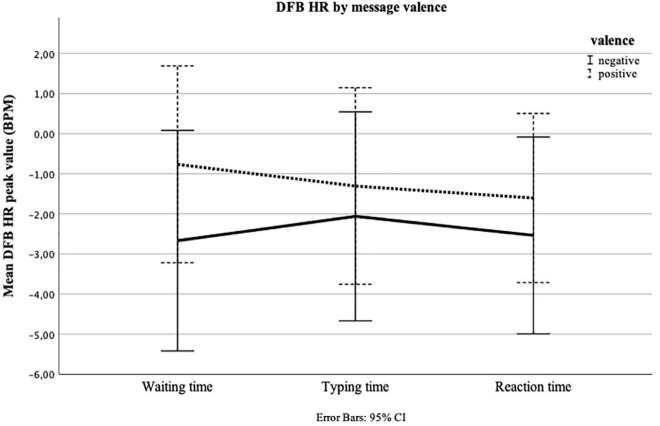
Repeated measures of heart rate (HR) in beats per minute (BPM) for Waiting time, Typing time, and Reaction time by message valence (positive = positive answer from peer; negative = negative answer from peer). The *y*-axis shows the mean peak value calculated as a deviation from the baseline measure (DFB) for the final 30 s of Waiting time, the first 30 s of Typing time, and the first 30 s of Reaction time.

**FIGURE 6:**
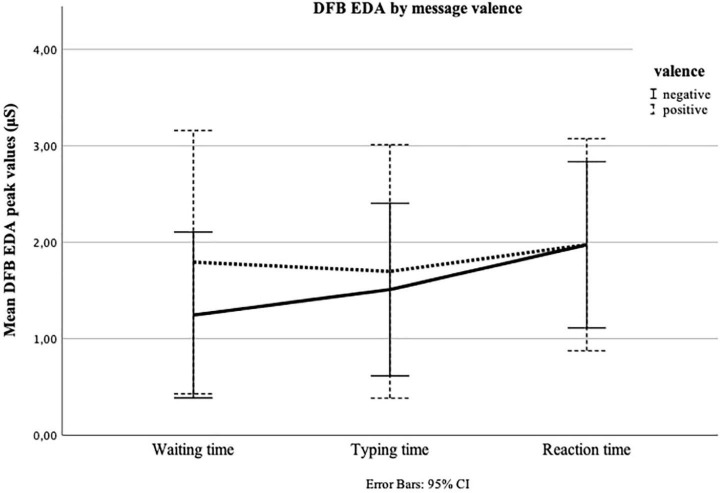
Repeated measures of electrodermal activity (EDA) in microSiemens (μS) for Waiting time, Typing time, and Reaction time by message valence (positive = positive answer from peer; negative = negative answer from peer). The *y*-axis shows the mean peak value calculated as a deviation from the baseline measure (DFB) for the final 30 s of Waiting time, the first 30 s of Typing time, and the first 30 s of Reaction time.

In the third repeated measures ANOVA, we looked at the change of DFB HR over time, considering message valence. The test for within-subjects effects showed that, across both conditions, i.e., positive and negative, HR once again did not change significantly over time [*F*(2,62) = 1.022, *p* = 0.349, $\eta_{\text{p}}^{2}$ = 0.016]. Neither was there a significant time × experimental group interaction [*F*(2,62) = 2.127, *p* = 0.134, $\eta_{\text{p}}^{2}$ = 0.033] (Figure 5). We repeated the ANOVA for the last time, now focusing on DFB EDA. The test of within-subjects effects showed that, across both conditions, i.e., positive and negative, EDA significantly changed over time [*F*(2,62) = 4.415, *p* = 0.026, $\eta_{\text{p}}^{2}$ = 0.065]. Yet, the change was not conditioned by message valence [*F*(2,62) = 1.462, *p* = 0.237, $\eta_{\text{p}}^{2}$ = 0.023]. The test of within-subjects contrasts [*F*(1,63) = 4.610, *p* = 0.036], and the examination of the estimated marginal means (Figure 6), indicated a linear increase in EDA, especially between Typing time and Reaction time. We reran all analyses, excluding the three outliers, but the results did not change.

We eventually conducted an independent samples *t*-test to examine possible differences in the self-report evaluation of the IM situation and the fictitious peer for message latency and message valence. For message latency, there was no significant difference in either of the two outcome variables; *t*_IM_(63) = −0.177, *p* = 0.860, *t*_peer_(63) = 0.710, *p* = 480. For message valence, we found a significant difference in both self-report measures; *t*_IM_(63) = −0.10.852, *p* < 0.001, *t*_peer_(63) = 7.188, *p* < 0.001. Positive messages were evaluated as more pleasant (*M* = 5.27, *SD* = 1.50) and the peer as more available (*M* = 4.55, *SD* = 1.33) than negative messages (*M* = 1.83, *SD* = 1.03) and their sender (*M* = 2.51, *SD* = 0.94). This finding confirms that the correct manipulation of message valence.

## Discussion

Instant messaging through smartphone-based messenger services like WhatsApp is increasingly used to interact on a daily basis, especially among younger populations. However, little is known about the emotional experience during IM communication. To shed light on the underlying experiential processes, the present experimental study incorporated psychophysiological data such as HR and EDA in the context of IM communication. We considered different yet typical affordances of IM communication: message latency and message valence to answer two research questions drawing from the Circumplex Model of Affect (Russell, 1980) on the emotional experience of synchronous vs. delayed and positive vs. negative messages. More precisely, we explored changes in the emotional experience – measured with HR and EDA levels for arousal and self-report perceptions for perceived valence – as individuals waited for, received, and processed incoming messages on the smartphone. The Circumplex Model of Affect assumes that external stimuli increase HR and EDA as a function of emotional arousal due to the activation of the SNS (Potter and Bolls, 2012). While we did not find significantly higher levels of HR during IM, we found an increase in EDA levels at the moment when participants received an answer from a fictitious peer, especially in the delayed condition. In other words, when individuals had to wait for a message for 7 min while not engaging in any other distracting activities, they were particularly aroused by the incoming message, independent of its valence. This result highlights three important points.

First, *the timing of IM communication matters*. More precisely, waiting for an anticipated response leads to even higher arousal when the response arrives. Like any social interaction, IM is characterized by individuals’ need for and anticipation of social rewards (Krach et al., 2010; Veissière and Stendel, 2018). However, since these rewards may not be immediate in the context of IM, they may create a state of increased emotional arousal, especially when the rewards (i.e., the response) eventually arrive in the form of notifications on the smartphone. Prior research has already shown that arousal induced by smartphone notifications is different from arousal due to arbitrary stimuli in the environment (Fortin et al., 2019). As individuals are notified about incoming messages, “the dopaminergic reward circuit [gets activated], leading the user to anticipate and seek these [social] rewarding notifications” (Veissière and Stendel, 2018, p. 4). According to the same authors, dopamine release causes emotional arousal, which is highest when the stimulus comes in (e.g., the peak in the first 30 s of reaction time that we considered in the present study).

In the present experiment, the waiting time was fixed to 7 min. However, we should not forget that IM is characterized by high variability in message latency. Individuals engaging in IM have only limited means to know (e.g., through the “is typing”-notification in WhatsApp chats) when the communication partner responds. Since people often expect fast responses when initiating IM conversations (Pielot et al., 2014), a lack of quick answers creates tensions and, therefore, may be the reason why people tend to check their smartphones more frequently for notifications of incoming messages. We found a significant increase in EDA from Typing time to Reaction time in both conditions, i.e., when the incoming message was either positive or negative. Based on our study results, we also conclude that the lack of quick responses creates emotional arousal that is not necessarily a sign of tensions but may equally be a sign of excitement and searched gratification, leading to a frequent checking of incoming notifications. In fact, the heightened emotional arousal in the delayed condition also holds when considering message valence.

Whether the experienced emotional arousal is a sign of distress or excitement requires further information that can be obtained through self-report evaluations of the IM situations. This brings us to the second important point: *Objective (i.e., physiological) and subjective (i.e., self-report) data convey different information on the emotional experience during different phases of IM.* Thus, they enrich each other in the interpretation of emotional experiences in mediated communication. Based on participants’ self-report evaluation of the IM situation and the involved peer, we found that message valence, but not message latency, predicted a differential evaluation of the IM situation. More precisely, positive messages were considered as more pleasant and their sender as more available than negative messages and their sender. This confirms findings from our prior experiment manipulating IM valence and latency (Petrocchi et al., 2020), which found that positive messages increased the subjective social presence, which, in turn, was positively related to a favorable perception of the peer who sent the messages. No such mediating effect was found for synchronous compared to delayed messages. This raises the question of why delayed messages are not evaluated as less pleasant than an immediate response. Recalling what we have just said about the gratifying experience of waiting for a message response, it may be that the discordant personal experiences rule each other out and thus lead to neither a positive nor a negative evaluation of the delayed IM situation.

The third and final point regards *methodological and theoretical considerations to improve future studies of the effects in Human–Computer-Interaction*. Among HR and EDA, the latter proved to be a better indicator of the SNS activation. In fact, HR can either increase or decrease as a sign of arousal, resulting in no arousal when averaging across participants of the same experimental condition. That said, future studies on the experience and consequences of IM, and Human–Computer-Interaction in general, should incorporate EDA as an indicator of emotional arousal and a predictor of studied outcomes. Technological advancements and the fast diffusion of wearable tracking devices may ease the study of physiological responses beyond self-report. Since increased EDA levels only indicate heightened arousal, additional data sources are needed to interpret the type of emotional response (i.e., distress, tension, or frustration vs. pleasure or excitement) as explicated in the Circumplex Model of Affect. These sources can be the Facial Action Coding System, electromyography, automatic face recognition systems, but also self-report measures (Mauss and Robinson, 2009; Wolf, 2015). The combination of objective and subjective data requires a solid theoretical basis incorporating psychophysiological measures in the context of mediated communication. Theoretical advancements in this regard are lacking to date but can be included, for example, in the Differential Susceptibility to Media Effects Model (Valkenburg and Peter, 2013), which specifies cognitive and emotional response states as antecedents of media effects. Eventually, Ecological Momentary Assessments (Shiffman et al., 2008), triggered by notifications on the smartphone, can be used to measure people’s immediate emotional response, i.e., perceived valence, related to incoming messages. This requires a paradigm shift away from a highly controlled experimental setting to measure emotional arousal due to a specific stimulus, given that the everyday life characterized by multiple stimuli limits conclusions on the physiological response to very specific stimuli of interest to the researcher.

Furthermore, the inclusion of additional self-report measures would allow investigating possible mediators and moderators of the physiological correlates. In this way, it would be possible to detect the emotional experience of individuals that are more vulnerable to problematic usage behaviors. Indeed, the rewarding experience of delayed gratifications likely results in repeated checking of incoming notifications, which is a sign of decreased inhibitory control, potentially contributing to problematic smartphone use (Busch and McCarthy, 2021) and experiences of phantom ringing and vibrations (Kruger and Djerf, 2017). For example, a study with a specific focus on IM has identified attachment anxiety as a risk factor (Kruger and Djerf, 2016). More precisely, “individuals who sought reassurance of their partner’s interests in their relationship were more likely to experience phantom ringing” (p. 58). Other potential risk factors are neuroticism (Marciano et al., 2020) and social anxiety (Annoni et al., 2021). Neurotic and socially anxious people are particularly concerned about negative evaluations, and a delay in the IM response may lead them to experience negative emotional arousal due to a perceived lack of interest from the communication partner indicative of a negative evaluation. Past research found that social anxiety is associated with physiological correlates in communication situations, though the association with HR is only moderate (Patterson and Ritts, 1997). Thus, personality risk factors and additional physiological measures should be considered as moderators in future studies investigating the emotional experience of message latency with physiological measures in the context of smartphone-based IM.

### Limitations and Future Directions

To the best of our knowledge, this is the first study investigating the emotional experience during IM by incorporating HR and EDA as two psychophysiological correlates and considering different phases of the communication situation from the Waiting time after sending out a request to the Reaction time just after receiving a response. While the real-time measurement of HR and EDA during IM is a major strength of the present study, some limitations need to be acknowledged. For example, the final sample size was small, also due to technical problems with the E4 wristband, which limited statistical power to detect minor effects of instant communication on psychophysiological reactions. Future studies with larger samples should replicate our study and compare conditional effects by simultaneously considering message valence and latency to shed light on the emotional response during IM communication. Larger samples also allow testing more complex models (e.g., mediation and moderation models) to study the indirect and conditional effects of IM on the subjective experience.

Furthermore, the data for this study stem from a laboratory experiment with an artificial IM context. Although such a design is typical in psychophysiological research (Potter and Bolls, 2012) to control for a variety of confounding variables, including environmental distractors and changes in light, noise, and temperature, it comes at the expense of ecological validity that allows generalizing the results to real-life settings, where IM communication is embedded in a variety of online and offline activities. A solution to this dilemma is the previously mentioned in-built sensors of smartphones, smartwatches, Ecological Momentary Assessments, and fitness trackers that are increasingly common and used in the population for self-monitoring of one’s activities and health. The data captured through these devices can be matched with the instant communication history to answer research questions on the emotional experience, including more subconscious aspects related to the psychophysiological response. The use of real-life known communication partners also allows detecting physiological reactions that are diminished or even absent when messaging with a fictitious peer. Our non-significant and small significant findings, in fact, may reflect a diminished perception of social judgment and reward by young adults who were aware of the hypothetical IM situation.

## Conclusion

The aim of this study was to investigate the emotional experience during IM by measuring emotional arousal through HR and EDA in addition to perceived valence through self-reports and by manipulating message latency and message valence. While we found no change in HR during the different measurement points (i.e., Waiting time, Typing time, Reaction time), we found an increase in EDA from Typing time to Reaction time, especially in the delayed condition where participants had to wait for 7 min before the IM partner started typing a response. EDA increased between Typing time and Reaction time independently of whether the response was positive or negative. These findings shed light on the psychophysiological processes underlying the anticipation of social reward in the context of incoming notifications in smartphone-mediated communication.

## Data Availability Statement

The raw data supporting the conclusions of this article can be found in the OSF repository https://osf.io/c26hg/.

## Ethics Statement

This study involving human participants was reviewed and approved by the Ethics Committee of the Università della Svizzera Italiana. All participants provided their written informed consent to participate in this study. The individual(s) provided their written informed consent for the publication of any identifiable images or data presented in this article.

## Author Contributions

A-LC: conceptualization, methodology, formal analysis, writing – original draft preparation, supervision, and funding acquisition. LM: conceptualization, methodology, formal analysis, supervision, and writing – review and editing. AA: investigation, data curation, formal analysis, and project administration. AO: conceptualization, methodology, data curation, formal analysis, and writing – review and editing. SP: conceptualization, methodology, supervision, and funding acquisition. All authors contributed to the article and approved the submitted version.

## Conflict of Interest

The authors declare that the research was conducted in the absence of any commercial or financial relationships that could be construed as a potential conflict of interest.

## Publisher’s Note

All claims expressed in this article are solely those of the authors and do not necessarily represent those of their affiliated organizations, or those of the publisher, the editors and the reviewers. Any product that may be evaluated in this article, or claim that may be made by its manufacturer, is not guaranteed or endorsed by the publisher.

## Footnotes

1
www.emojitracker.com

2
www.empatica.com
